# Supplementary material for: Internal Validation of the ASFV MONODOSE dtec-qPCR Kit for African Swine Fever Virus Detection under the UNE-EN ISO/IEC 17025:2005 Criteria
Source: Vet Sci. 2023 Sep 8;10(9):564. doi: 10.3390/vetsci10090564 (PMC10535882; doi:10.3390/vetsci10090564)
Supplement: Supplementary file 1 [file vetsci-10-00564-s001.zip › vetsci-2517710-supplementary.pdf]

Table S1. Diagnostic analysis results (Cts) for African swine fever virus qPCR.

ID	Sample	Isolate	Origin	Host	ASFV GPS™-mix	King et al.	Fernández Pinero et al.
1	Blood serum	E70	Spain	Domestic pig	29.98	32.72	31.49
2	Blood serum	BF07 OUAGA 2	Burkina	Domestic pig	32.53	34.84	35.79
3	Blood serum	SS14/WB-Sassari1	Italy	Wild boar	31.83	33.35	32.33
4	Blood serum	SS14/DP-Cagliari1	Italy	Domestic pig	36.18	Negative	36.67
5	Blood serum	Arm07	Armenia	Domestic pig	29.68	31.56	30.51
6	Blood serum	Ukr12/Zapo	Ukraine	Domestic pig	28.30	30.79	28.48
7	Blood serum	Ukr15/DP-Kieve 1	Ukraine	Domestic pig	32.43	35.57	34.28
8	Blood serum	LT14/1490	Lithuania	Wild boar	29.89	32.01	27.41
9	Blood serum	Pol14/Krus	Poland	Wild boar	30.64	33.12	30.75
10	Blood serum	Lv14/DP/Robez3	Latvia	Domestic pig	33.59	35.7	35.9
11	Blood serum	Est14/WB-Valga-1	Estonia	Wild boar	32.58	34.85	35.22
12	Blood serum	Est15/WB-Tartu14	Estonia	Wild boar	35.32	35.86	37.39
13	Blood serum	MOL16/DP-CERNO1	Moldova	Domestic pig	35.71	Negative	32.63
14	Blood serum	MOL16/DP-MOSA1	Moldova	Domestic pig	33.76	36	34.47
15	Blood serum	Moz64	Mozambique	Domestic pig	29.99	32.71	32.64
16	Blood serum	MwLil 20/1	Malawi	Tick	33.08	35.09	35.18
17	Blood serum	Ken11/KisP52	Kenia	Domestic pig	34.76	32.67	33.65
18	Blood serum	Ken06.Bus	Kenia	Domestic pig	42.53	Negative	39.65
19	Blood serum	Ken08Tk.2/1	Kenia	Tick	35.54	34.22	32.46
20	Blood serum	UG10/Tk3.2	Uganda	Tick	38.88	39.4	37.96
21	Blood serum	Eth13/1505	Ethiopia	Domestic pig	32.37	34.58	34.56
22	Blood serum	-	Spain	Domestic pig	Negative	Negative	Negative
23	Blood serum	-	Spain	Domestic pig	Negative	Negative	Negative
24	Blood serum	-	Spain	Domestic pig	Negative	Negative	Negative

25	Blood serum	-	Spain	Domestic pig	Negative	Negative	Negative
26	Blood serum	-	Spain	Domestic pig	Negative	Negative	Negative
27	Blood serum	-	Spain	Domestic pig	Negative	Negative	Negative
28	Blood serum	-	Spain	Domestic pig	Negative	Negative	Negative
29	Blood serum	-	Spain	Domestic pig	Negative	Negative	Negative
30	Blood serum	-	Spain	Domestic pig	Negative	Negative	Negative
31	Blood serum	-	Spain	Domestic pig	Negative	Negative	Negative
32	Blood serum	-	Spain	Domestic pig	Negative	Negative	Negative
33	Blood serum	-	Spain	Domestic pig	Negative	Negative	Negative
34	Blood serum	-	Spain	Domestic pig	Negative	Negative	Negative
35	Blood serum	-	Spain	Domestic pig	Negative	Negative	Negative
36	Blood serum	-	Spain	Domestic pig	Negative	Negative	Negative
37	Blood serum	-	Spain	Domestic pig	Negative	Negative	Negative
38	Blood serum	-	Spain	Domestic pig	Negative	Negative	Negative
39	Blood serum	-	Spain	Domestic pig	Negative	Negative	Negative
40	Blood serum	-	Spain	Domestic pig	Negative	Negative	Negative
41	Blood serum	-	Spain	Domestic pig	Negative	Negative	Negative
42	Blood serum	-	Spain	Domestic pig	Negative	Negative	Negative
43	Blood serum	-	Spain	Domestic pig	Negative	Negative	Negative
44	Blood serum	-	Spain	Domestic pig	Negative	Negative	Negative
45	Blood serum	-	Spain	Domestic pig	Negative	Negative	Negative
46	Blood serum	-	Spain	Domestic pig	Negative	Negative	Negative
47	Blood serum	-	Spain	Domestic pig	Negative	Negative	Negative
48	Blood serum	-	Spain	Domestic pig	Negative	Negative	Negative
49	Blood serum	-	Spain	Domestic pig	Negative	Negative	Negative
50	Blood serum	-	Spain	Domestic pig	Negative	Negative	Negative
51	Blood serum	-	Spain	Domestic pig	Negative	Negative	Negative
52	Blood serum	-	Spain	Domestic pig	Negative	Negative	Negative
53	Blood serum	-	Spain	Domestic pig	Negative	Negative	Negative

54	Blood serum	-	Spain	Domestic pig	Negative	Negative	Negative
55	Blood serum	-	Spain	Domestic pig	Negative	Negative	Negative
56	Blood serum	-	Spain	Domestic pig	Negative	Negative	Negative
57	Blood serum	-	Spain	Domestic pig	Negative	Negative	Negative
58	Blood serum	-	Spain	Domestic pig	Negative	Negative	Negative
59	Blood serum	-	Spain	Domestic pig	Negative	Negative	Negative
60	Blood serum	-	Spain	Domestic pig	Negative	Negative	Negative
61	Blood serum	-	Spain	Domestic pig	Negative	Negative	Negative
62	Kidney	E70	Spain	Domestic pig	28.38	31.42	31.43
63	Kidney	BF07 OUAGA 2	Burkina	Domestic pig	31.46	33.97	29.83
64	Kidney	SS14/WB-Sassari1	Italy	Wild boar	29.81	33.53	32.6
65	Kidney	SS14/DP-Cagliari1	Italy	Domestic pig	33.74	39.34	35.47
66	Kidney	Arm07	Armenia	Domestic pig	27.88	31.46	31.05
67	Kidney	Ukr12/Zapo	Ukraine	Domestic pig	27.47	30.26	28.92
68	Kidney	Ukr15/DP-Kieve 1	Ukraine	Domestic pig	29.87	33.19	32.57
69	Kidney	LT14/1490	Lithuania	Wild boar	27.60	30.66	29.01
70	Kidney	Pol14/Krus	Poland	Wild boar	29.16	31.40	31.71
71	Kidney	Lv14/DP/Robez3	Latvia	Domestic pig	32.02	34.69	33.87
72	Kidney	Est14/WB-Valga-1	Estonia	Wild boar	31.43	34.20	31.88
73	Kidney	Est15/WB-Tartu14	Estonia	Wild boar	32.56	36.96	33.38
74	Kidney	MOL16/DP-CERNO1	Moldova	Domestic pig	33.14	36.19	33.42
75	Kidney	MOL16/DP-MOSA1	Moldova	Domestic pig	31.86	35.92	34.91
76	Kidney	Moz64	Mozambique	Domestic pig	28.26	31.13	31.31
77	Kidney	MwLil 20/1	Malawi	Tick	31.95	34.72	34.3
78	Kidney	Ken11/KisP52	Kenia	Domestic pig	32.78	31.94	31.02
79	Kidney	Ken06.Bus	Kenia	Domestic pig	37.75	37.44	35.14
80	Kidney	Ken08Tk.2/1	Kenia	Tick	31.56	30.37	29.13
81	Kidney	UG10/Tk3.2	Uganda	Tick	38.26	37.66	37.63
82	Kidney	Eth13/1505	Ethiopia	Domestic pig	29.57	33.90	26.61

83	Kidney	-	Spain	Domestic pig	Negative	Negative	Negative
84	Kidney	-	Spain	Domestic pig	Negative	Negative	Negative
85	Kidney	-	Spain	Domestic pig	Negative	Negative	Negative
86	Kidney	-	Spain	Domestic pig	Negative	Negative	Negative
87	Kidney	-	Spain	Domestic pig	Negative	Negative	Negative
88	Kidney	-	Spain	Domestic pig	Negative	Negative	Negative
89	Kidney	-	Spain	Domestic pig	Negative	Negative	Negative
90	Kidney	-	Spain	Domestic pig	Negative	Negative	Negative
91	Kidney	-	Spain	Domestic pig	Negative	Negative	Negative
92	Heart	E70	Spain	Domestic pig	28.00	Neg-Inhib IC	30.1
93	Heart	BF07 OUAGA 2	Burkina	Domestic pig	31.27	34.61	31.88
94	Heart	SS14/WB-Sassari1	Italy	Wild boar	30.46	32.81	31.68
95	Heart	SS14/DP-Cagliari1	Italy	Domestic pig	34.98	Negative	35.09
96	Heart	Arm07	Armenia	Domestic pig	28.54	30.83	29.72
97	Heart	Ukr12/Zapo	Ukraine	Domestic pig	29.22	29.9	29.98
98	Heart	Ukr15/DP-Kieve 1	Ukraine	Domestic pig	30.1	32.44	30.45
99	Heart	LT14/1490	Lithuania	Wild boar	28.13	30.11	29.37
100	Heart	Pol14/Krus	Poland	Wild boar	28.79	31.51	30.77
101	Heart	Lv14/DP/Robez3	Latvia	Domestic pig	31.88	34.57	33.04
102	Heart	Est14/WB-Valga-1	Estonia	Wild boar	31.66	34.16	32.87
103	Heart	Est15/WB-Tartu14	Estonia	Wild boar	33.20	35.12	35.5
104	Heart	MOL16/DP-CERNO1	Moldova	Domestic pig	32.27	41.52	38.2
105	Heart	MOL16/DP-MOSA1	Moldova	Domestic pig	31.84	35.08	34.53
106	Heart	Moz64	Mozambique	Domestic pig	28.58	31.28	31
107	Heart	MwLil 20/1	Malawi	Tick	32.36	34.12	33.01
108	Heart	Ken11/KisP52	Kenia	Domestic pig	34.2	31.48	29.8
109	Heart	Ken06.Bus	Kenia	Domestic pig	36.18	34.16	33.72
110	Heart	Ken08Tk.2/1	Kenia	Tick	32.49	30.22	28.41

111	Heart	UG10/Tk3.2	Uganda	Tick	39.51	37.28	34.37
112	Heart	Eth13/1505	Ethiopia	Domestic pig	30.37	33.77	32.85
113	Heart	-	Spain	Domestic pig	Negative	Negative	Negative
114	Heart	-	Spain	Domestic pig	Negative	Negative	Negative
115	Heart	-	Spain	Domestic pig	Negative	Negative	Negative
116	Heart	-	Spain	Domestic pig	Negative	Negative	Negative
117	Heart	-	Spain	Domestic pig	Negative	Negative	Negative
118	Heart	-	Spain	Domestic pig	Negative	Negative	Negative
119	Heart	-	Spain	Domestic pig	Negative	Negative	Negative
120	Heart	-	Spain	Domestic pig	Negative	Negative	Negative
121	Heart	-	Spain	Domestic pig	Negative	Negative	Negative
122	Liver	E70	Spain	Domestic pig	28.00	Neg-Inhib IC	30.1
123	Liver	BF07 OUAGA 2	Burkina	Domestic pig	31.27	34.61	31.88
124	Liver	SS14/WB-Sassari1	Italy	Wild boar	30.46	32.81	31.68
125	Liver	SS14/DP-Cagliari1	Italy	Domestic pig	34.98	Negative	35.09
126	Liver	Arm07	Armenia	Domestic pig	28.54	30.83	29.72
127	Liver	Ukr12/Zapo	Ukraine	Domestic pig	29.22	29.9	29.98
128	Liver	Ukr15/DP-Kieve 1	Ukraine	Domestic pig	30.1	32.44	30.45
129	Liver	LT14/1490	Lithuania	Wild boar	28.13	30.11	29.37
130	Liver	Pol14/Krus	Poland	Wild boar	28.79	31.51	30.77
131	Liver	Lv14/DP/Robez3	Latvia	Domestic pig	31.88	34.57	33.04
132	Liver	Est14/WB-Valga-1	Estonia	Wild boar	31.66	34.16	32.87
133	Liver	Est15/WB-Tartu14	Estonia	Wild boar	33.20	35.12	35.5
134	Liver	MOL16/DP-CERNO1	Moldova	Domestic pig	32.27	41.52	38.2
135	Liver	MOL16/DP-MOSA1	Moldova	Domestic pig	31.84	35.08	34.53
136	Liver	Moz64	Mozambique	Domestic pig	28.58	31.28	31
137	Liver	MwLil 20/1	Malawi	Tick	32.36	34.12	33.01
138	Liver	Ken11/KisP52	Kenia	Domestic pig	34.2	31.48	29.8

139	Liver	Ken06.Bus	Kenia	Domestic pig	36.18	34.16	33.72
140	Liver	Ken08Tk.2/1	Kenia	Tick	32.49	30.22	28.41
141	Liver	UG10/Tk3.2	Uganda	Tick	39.51	37.28	34.37
142	Liver	Eth13/1505	Ethiopia	Domestic pig	30.37	33.77	32.85
143	Liver	-	Spain	Domestic pig	Negative	Negative	Negative
144	Liver	-	Spain	Domestic pig	Negative	Negative	Negative
145	Liver	-	Spain	Domestic pig	Negative	Negative	Negative
146	Liver	-	Spain	Domestic pig	Negative	Negative	Negative
147	Liver	-	Spain	Domestic pig	Negative	Negative	Negative
148	Liver	-	Spain	Domestic pig	Negative	Negative	Negative
149	Liver	-	Spain	Domestic pig	Negative	Negative	Negative
150	Liver	-	Spain	Domestic pig	Negative	Negative	Negative
151	Liver	-	Spain	Domestic pig	Negative	Negative	Negative
152	Tonsil	E70	Spain	Domestic pig	28.51	30.73	27.47
153	Tonsil	BF07 OUAGA 2	Burkina	Domestic pig	30.91	35.77	35.69
154	Tonsil	SS14/WB-Sassari1	Italy	Wild boar	29.86	34.12	31.16
155	Tonsil	SS14/DP-Cagliari1	Italy	Domestic pig	32.64	36.12	34.24
156	Tonsil	Arm07	Armenia	Domestic pig	28.14	31.81	30.26
157	Tonsil	Ukr12/Zapo	Ukraine	Domestic pig	27.47	30.42	29.66
158	Tonsil	Ukr15/DP-Kieve 1	Ukraine	Domestic pig	29.98	33.3	32.2
159	Tonsil	LT14/1490	Lithuania	Wild boar	28.21	31.2	29.67
160	Tonsil	Pol14/Krus	Poland	Wild boar	28.70	32.16	31.56
161	Tonsil	Lv14/DP/Robez3	Latvia	Domestic pig	31.71	35.54	35.3
162	Tonsil	Est14/WB-Valga-1	Estonia	Wild boar	31.87	35.17	32.61
163	Tonsil	Est15/WB-Tartu14	Estonia	Wild boar	32.64	36.76	36.54
164	Tonsil	MOL16/DP-CERNO1	Moldova	Domestic pig	33.31	38.51	37.38
165	Tonsil	MOL16/DP-MOSA1	Moldova	Domestic pig	31.20	35.46	35
166	Tonsil	Moz64	Mozambique	Domestic pig	28.11	31.27	31.02
167	Tonsil	MwLil 20/1	Malawi	Tick	31.82	34.93	33.86

168	Tonsil	Ken11/KisP52	Kenia	Domestic pig	33.10	32.36	30.87
169	Tonsil	Ken06.Bus	Kenia	Domestic pig	36.68	37.31	35.27
170	Tonsil	Ken08Tk.2/1	Kenia	Tick	32.24	31.15	29.14
171	Tonsil	UG10/Tk3.2	Uganda	Tick	37.79	Negative	35.27
172	Tonsil	Eth13/1505	Ethiopia	Domestic pig	30.56	34.39	36.72
173	Tonsil	-	Spain	Domestic pig	Negative	Negative	Negative
174	Tonsil	-	Spain	Domestic pig	Negative	Negative	Negative
175	Tonsil	-	Spain	Domestic pig	Negative	Negative	Negative
176	Tonsil	-	Spain	Domestic pig	Negative	Negative	Negative
177	Tonsil	-	Spain	Domestic pig	Negative	Negative	Negative
178	Tonsil	-	Spain	Domestic pig	Negative	Negative	Negative
179	Tonsil	-	Spain	Domestic pig	Negative	Negative	Negative
180	Tonsil	-	Spain	Domestic pig	Negative	Negative	Negative
181	Tonsil	-	Spain	Domestic pig	Negative	Negative	Negative
